# Supplementary material for: Comparison of Efavirenz and Doravirine Developmental Toxicity in an Embryo Animal Model
Source: Int J Mol Sci. 2023 Jul 19;24(14):11664. doi: 10.3390/ijms241411664 (PMC10380689; doi:10.3390/ijms241411664)
Supplement: Supplementary file 1 [file ijms-24-11664-s001.zip › ijms-2494165-supplementary.pdf]

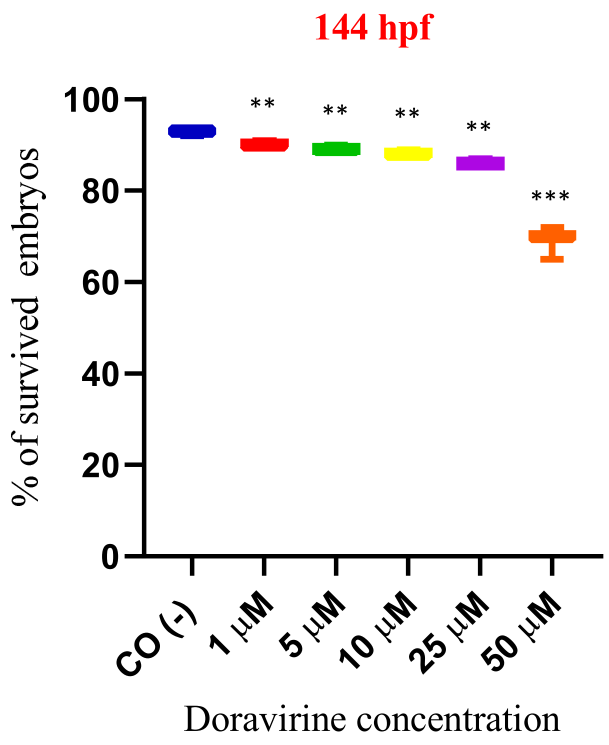

Figure S1. Survival rate of dechorionated zebrafish embryos at 144 hours post fertilization (hpf) after exposure to doravirine (DOR) at gastrula stage (4 hpf) by the immersion method. CO (-) represents embryos exposed to drug solvent only (fish water plus 0.1% dimethyl sulfoxide, DMSO). X-axis shows drug doses used for exposure of embryos; Y-axis shows the corresponding survival percentages. Results are expressed as mean ± SD of three independent experiments, with 30 embryos for each experiment and each treatment. (** $p < 0.005$ vs. control group; *** $p < 0.001$ vs. control group)

Table S1. Primers used in RT-PCR experiments.

gene	primer sequence
pparg for	CCTGTCCGGGAAGACCAGCG
pparg rev	GTGCTCGTGGAGCGGCATGT
srebf1 for	CATCCACATGGCTCTGAGTG
srebf1 rev	CTCATCCACAAAGAAGCGGT
cebpa for	AACGGAGCGAGCTTGACTT
cebpa rev	AAATCATGCCCATTAGCTGC
rpl13a for	TCTGGAGGACTGTAAGAGGTATGC
rpl13a rev	AGACGCACAATCTTGAGAGCAG
